# Incidence of idiopathic nephrotic syndrome during the Covid-19 pandemic in the Paris area (France) and in the Netherlands

**DOI:** 10.1007/s00467-023-06006-9

**Published:** 2023-05-16

**Authors:** Floor Veltkamp, Victoire Thenot, Carlijn Mussies, Bas van Lieshout, Hessel Peters-Sengers, Jesper Kers, Djera H. Khan, Julien Hogan, Sandrine Florquin, Antonia H. M. Bouts, Claire Dossier, on behalf of the NEPHROVIR network, the LEARNS consortium

**Affiliations:** 1 Department of Pediatric Nephrology, Emma Children’s Hospital, Amsterdam University Medical Centers, University of Amsterdam, Meibergdreef 9, Amsterdam, 1109 AZ The Netherlands; 2 Department of Pediatric Nephrology, Robert-Debré Hospital, APHP, Paris, France; 3 Center for Experimental and Molecular Medicine, Amsterdam University Medical Centers, University of Amsterdam, Amsterdam, The Netherlands; 4 Department of Epidemiology and Data Science, VU University Amsterdam, Amsterdam University Medical Center, Amsterdam, Netherlands; 5 Department of Pathology, Amsterdam University Medical Centers, University of Amsterdam, Amsterdam, The Netherlands; 6 Department of Pathology, Leiden University Medical Center, Leiden, The Netherlands; 7 Van ‘t Hoff Institute for Molecular Sciences, University of Amsterdam, Amsterdam, The Netherlands

**Keywords:** Nephrotic syndrome, Paediatric, Incidence, Covid-19

## Abstract

**Background:**

The aetiology of idiopathic nephrotic syndrome (INS) remains partially unknown. Viral infections have been associated with INS onset. Since we observed fewer first onset INS cases during the Covid-19 pandemic, we hypothesised that lower INS incidence was the result of lockdown measures. Therefore, the aim of this study was to evaluate the incidence of childhood INS before and during the COVID-19 pandemic in two independent European INS cohorts.

**Methods:**

Children with new INS in the Netherlands (2018–2021) and Paris area (2018–2021) were included. We estimated incidences using census data for each region. Incidences were compared using two proportion Z-tests.

**Results:**

A total of 128 and 324 cases of first onset INS were reported in the Netherlands and Paris area, respectively, corresponding to an annual incidence of 1.21 and 2.58 per 100,000 children/year. Boys and young children (< 7 years) were more frequently affected. Incidence before and during the pandemic did not differ. When schools were closed, incidence was lower in both regions: 0.53 vs. 1.31 (*p =* 0.017) in the Netherlands and 0.94 vs. 2.63 (*p =* 0.049) in the Paris area. During peaks of hospital admissions for Covid-19, no cases were reported in the Netherlands or Paris area.

**Conclusions:**

Incidence of INS before and during the Covid-19 pandemic was not different, but when schools were closed during lockdown, incidence was significantly lower. Interestingly, incidences of other respiratory viral infections were also reduced as was air pollution. Together, these results argue for a link between INS onset and viral infections and/or environmental factors.

**Graphical abstract:**

**Figure:**
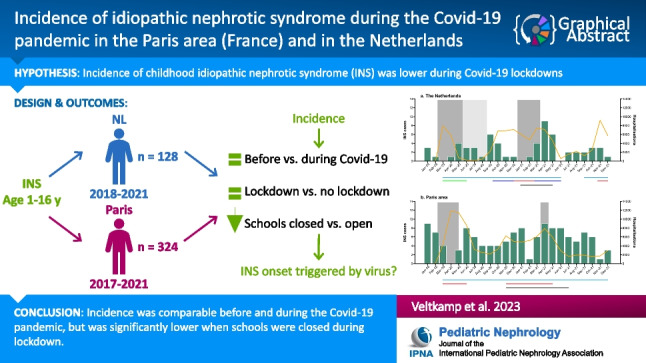
A higher resolution version of the Graphical abstract is available as Supplementary information

**Supplementary information:**

The online version contains supplementary material available at 10.1007/s00467-023-06006-9.

## Introduction

Idiopathic nephrotic syndrome (INS) is the most common glomerulopathy in paediatric nephrology, but is a rare disease in the general paediatric population. Its incidence is estimated to be between 1.2 and 16.9 per 100,000 children per year [1–3], varying widely between global regions and ethnicities [4]. In the Netherlands, between 2007 and 2009, the incidence of INS was estimated to be 1.52 per 100,000 children per year, which corresponds with ± 60 cases annually [5]. In the Paris area, a higher incidence of 3.35 per 100,000 children per year (± 75 cases annually) was found between December 2007 and May 2010 [6].

Patients with new onset INS present with profound oedema, heavy proteinuria (urinary protein-to-creatinine ratio (uPCR) > 200 mg /mmol), and hypoalbuminaemia (serum albumin < 25 g/L) [7]. The exact pathogenesis is still largely unknown, but different theories have been proposed. First, the immune system seems to play an important role in the aetiology of INS, which involves dysfunction or dysregulation of T and B cells. The T cell hypothesis was first proposed by Shalboub in 1974 [8]. Later, involvement of B cells became apparent following sustained remission of the disease in patients receiving rituximab [9] and the recent identification of autoantibodies (UCHL1, anti Nephrin) [10, 11]. Second, the presence of a circulating permeability factor plays a role, particularly in focal segmental glomerulosclerosis [12]. To date, this factor has yet to be identified. Third, in line with the T cell hypothesis, viral pathogens or allergens may trigger the onset of INS as a two-hit mechanism by inducing CD80 in the podocytes, which cannot be effectively cleared due to a dysfunctional T cell response [13, 14]. Direct evidence has never been shown, however, epidemiological data support the association between viral triggers (Epstein-Barr virus (EBV) and enterovirus) and INS onset [14, 15].

When the world was hit by the rapidly emerging and newly discovered coronavirus (SARS-CoV-2) causing a global pandemic [16], governments across the world implemented lockdown measures to contain the spread of the virus. Lockdown measures also impacted children and adolescents, but varied strongly in degree, timing, and duration between countries. Measures included the closing of schools, sports events, and non-essential shops, as well as group size restrictions, the obligation to wear a facemask, and travel restrictions (both locally and internationally).

During these periods of lockdown, study teams of the LEARNS (the Netherlands) and NEPHROVIR-3 (Paris area, France) trial noticed a decrease in the number of first onset INS, hypothesising that this may be due to less exposure to viral pathogens as a – desired – result of social distancing. This phenomenon has previously been suggested for relapse rate [17–19]. Therefore, the aim of this study is to compare the incidence of INS before (before March, 2020) and during (from March, 2020 to December, 2021) the Covid-19 pandemic in the Netherlands and the Paris area. Additionally, the impact of different lockdown measures on incidence of INS was studied. To do so, we used incidence data from the LEARNS study and NEPHROVIR-3 study.

## Methods

### Study design

We conducted two separate, retrospective, cross-sectional, observational studies to estimate the incidence of INS in the Netherlands (January 2018 – December 2021) and the Paris area (January 2017 – December 2021). All new onset INS cases were prospectively registered as part of the ongoing clinical trials. In the Netherlands, an online survey was sent to all hospitals with a paediatric ward by email in order not to miss cases. After two and four weeks, a reminder was sent. An overview of all contacted hospitals can be found in the Online Resource 1. The survey was available online in Google Forms and consisted of 9 items divided over two parts: respondent and case details (see Online Resource 2). All hospitals participating in the NEPHROVIR-3 study were required to report any INS case during the recruitment phase, but since recruitment ended in February, 2020, all sites were contacted to report cases until December, 2021.

### Study population

All children between 1 and 16 years of age who presented with a new onset INS in the Netherlands or Paris area were screened for eligibility for inclusion in either the LEARNS or the NEPHROVIR-3 study, respectively. INS was defined as the presence of oedema, proteinuria (uPCR > 200 mg/mmol), and hypoalbuminaemia (serum albumin < 25 g/L). Children with non-idiopathic nephrotic syndrome (secondary, genetic, or congenital) or children not living in either the Netherlands or Paris area were excluded. The population at risk consists of all children aged 1–16 years living in the Netherlands or Paris area.

### Data collection

Date of first presentation, sex, and age were recorded on trial subject screening and enrolment logs. All data were screened for duplicates based on the case details. Census data (per January 1^st^) were extracted from the official statistic organs of the Netherlands (CBS: Centraal Bureau voor de Statistiek) and France (INSEE: Institut National de la Statistique et des Études Économiques).

To identify which and when lockdown measures were in effect, governmental and (national) news websites were used. Since testing policies changed over time in both countries, Covid-19-related hospitalisation, rather than confirmed cases of Covid-19, were used to illustrate the Covid-19 burden. Hospitalisation data were freely available from the website of the National Institute for Public Health and the Environment (*Dutch*: Rijksinstituut voor Volksgezondheid en Milieu (RIVM)) and Public Health France (*French*: Santé Publique France).

### Outcome measures

The primary outcome was the difference in incidence of INS between before (before March 2020) and during (from March 2020) the Covid-19 pandemic in the Netherlands and Paris area. Incidence was expressed as the number of new cases per 100,000 children (≤ 16 years) at risk per year. Secondary outcomes included the effect of different lockdown measures on incidence in the two regions separately.

### Statistical analysis

Descriptive continuous patient data are presented as the mean (standard deviation (SD)) or median (interquartile range (IQR)), depending on distribution. Discrete data are presented as frequencies and proportions (%).

To correct for missing cases, the true estimate was calculated. This estimate was based on the reported cases per month, adjusted for the total response rate for each survey. The response rate is the proportion of hospitals that completed the survey, independent of whether a case was reported. Assuming that missing reports were random and occurred independently from each other, the following equation was used:

$$True\;number\;of\;cases=\frac{Reported\;number\;of\;cases(n)}{Response\;rate(\%)}\times100$$

To calculate the confidence intervals for the true number of cases, a Monte Carlo simulation was performed (*n* = 1000 simulations), where reported cases followed a Poisson distribution and response rate a binomial distribution.

The incidence of INS in both regions was calculated by dividing the true number of cases by the number of children between 1 and 16 years of age living in the Netherlands or Paris area (per January 1^st^ of 2021) corrected for the total observation period:

$$Incidence\left(I\right)=\frac{True\;number\;of\;cases\;INS}{Children\;at\;risk}\times\frac{12}{Observation\;period\;(months)}\times100,000$$

Confidence intervals were obtained from the Monte Carlo simulation for the true number of cases estimate. Incidences (between Covid-period, sex, age group, lockdowns, and measures) were compared using a two-proportion Z-test. A *p*-value of < 0.05 was considered to be significantly different. All statistical tests were performed using R studio version 3.6.1 [20].

### Ethical approval

This study was performed in line with the principles of the Declaration of Helsinki. Approval was granted by the Ethics Committee of the Amsterdam University Medical Centers, location University of Amsterdam on March 8^th^, 2018 (2017_310). All subjects and/or their parents (if applicable) signed for informed consent. The LEARNS study is registered (www.trialregister.nl; NL6826). Further, the Ethics Committee concluded that the additional survey did not fall under scope of the Dutch Medical Research involving Humans Act (*Dutch:* Wet Medisch-Wetenschappelijk Onderzoek met mensen (WMO)) and a waiver of informed consent was obtained. The NEPHROVIR-3 study was approved by the Ethics Committee (*French:* Comité de Protection des Personnes) of Ile-De-France IV on January 26^th^, 2017 and registered (www.clinicaltrials.org, NCT02818738). For both studies, all subjects and/or their parents (if applicable) signed for informed consent. All survey data were collected anonymously.

## Results

A total of 118 and 324 unique cases were reported during the observation periods of the studies in the Netherlands (2018–2021) and the Paris area (2017–2021), respectively. The Dutch survey yielded an additional of 10 cases that were initially missed. Thus, 128 and 324 cases were included in the analysis (Table 1).

**Table 1:** Baseline characteristics of INS patient with a first onset between January 1^st^, 2018 and December 31^st^, 2021 in the nationwide cohort in The Netherlands and the regionwide cohort in the Paris area, France

Incidence characteristics	The Netherlands	Paris area, France	
Paediatric hospitals/wards	89	34	
Response, n (%)	80 (90%)^b^	34 (100%)	
Reported cases, n			
Total 2017 2018 2019 2020 2021	128 NA 40 29 28 33	324 65 59 69 61 65	
Population at risk^a^, n			
Total (1–16 years) Boys Girls < 7 years < 12 years	2,936,425 1,504,400 1,432,025 1,044,899 1,971,304	2,516,400 1,280,466 1,235,934 957,252 1,747,578	
Patient characteristics	The Netherlands	Paris area France	*p*-value
Age, years, median (IQR)	5.0 (3–8)	4.7 (3–7)	
Sex, n (%)			
Male	80 (62.5)	216 (66.7)	
Timing of first presentation^c^, n (%)			
Quarter 1 Quarter 2 Quarter 3 Quarter 4	27 (21.0) 37 (28.7) 32 (25.0) 32 (25.0)	81 (25) 82 (25.3) 75 (23.1) 86 (26.5)	
Covid-19 pandemic, n (%)			
Before During	73 (57.0) 55 (43.0)	211 (65.1) 113 (34.9)	
Lockdown measure, n (%)			< 0.001
Any Schools/Sports clubs closed Group size restrictions Facemask obligation	29 (60) 8 (16.5) 34 (61.8) 31 (56.4)	13 (11) 4 (3.5) – NA^d^	
SARS-CoV-2 infection, n (%)			
Positive Missing	1 (1.8) 30 (54.5)	NA NA	

^a^Population at risk at January 1^st^, 2021

^b^To correct for hospitals that did not respond to the survey but from which patients were referred to participating hospitals, an estimated 90% response rate was used for the Monte Carlo simulation

^c^Presentations in each quartile within the study period are summed. Quarter 1: January, February, March; Quarter 2: April, May, June; Quarter 3: July, August, September; Quarter 4: October, November, December

^d^Unable to classify due to changing restrictions per age group

Following the first cases and rapid spread of the virus, both countries implemented restrictive measures of varying degree. An overview and timeline of the measures can be found in Table 2. Fundamental hygiene rules applied for both countries during the pandemic: keep 1.5-m distance, wash your hands, stay at home and test yourself in case of symptoms, and ventilate rooms.

**Table 2:** Overview of lockdown measures in the Netherlands and the Paris area

	Netherlands		Paris area, France	
Lockdown measure	Date in effect	Specification	Date in effect	Specification
Basic rules	Mar 9^th^ 2020 – Dec 31^st^, 2021	Washing hands regularly Sneeze and cough in elbow Stay at home when ill Keep 1.5 meter distance Health pass (tested, vaccinated, or recovered)	Mar 17^th^, 2020 – Dec 31^st^, 2021	
Stay at home order	Mar 12^th^, 2020 – Jun 1^st^, 2020	Strongly advised to stay at home as much as possible, avoid social contacts, work from home, and not to travel.	Mar 17^th^, 2020 – Jun 2^nd^, 2020	Travel restricted to 1 hour within 1 km of home
			Oct 30^th^, 2020 – Dec 15^th^, 2020	Travel restricted to 3 hours within 20 km of home. Curfew between 21:00–06:00h
	Jan 25^th^, 2021 – Mar 30^th^, 2021	Curfew between 21:00–04:30h	Apr 3^rd^, 2021 – Apr 26^th^, 2021	Domestic travel ban, within 10 km of house, no time restriction. Curfew between 19:00–06:00h
	Mar 31^st^, 2021 – Apr 28^th^ 2021	Curfew between 22:00–04:30h	Jun 30^th^, 2021	Curfew lifted
Testing policy	Until Jun 1^st^, 2020	Testing only in hospitalised patients. In case of symptoms.		
	Jun 1^st^, 2020	In case of close contact		
	Dec 1^st^, 2020 – Dec 31^st^, 2021	Testing for entrance to restaurants, museums, and events		
Vaccination	Jan 6^th^ 2021	First vaccination	Dec 27^th^, 2020	First vaccination
	Nov 2^nd^ 2021	Booster vaccination available for the elderly and healthcare workers		
Schools	Mar 16^th^,2020 – Jun 8^th^, 2020	Closing primary schools: reopening at 50% capacity from May 11^th^	Mar 17^th^, 2020 – May 11^th^, 2020	Closing schools
	Mar 16^th^, 2020 – Jun 1^st^, 2020	Closing secondary schools	Apr 5^th^, 2021 – Apr 26^th^, 2021	Closing schools. Secondary and high schools reopened May 3^rd^, 2021.
	Dec 15^th^, 2020 – Feb 28^th^, 2021	Closing primary schools		
	Feb 15^th^, 2021 – May 31^st^, 2021	Closing secondary schools: reopening for ≥1 day/week from May 1^st^		
Bars and restaurants	Mar 15^th^, 2020 – Jun 1^st^, 2020	Closing all bars and restaurants	Mar 17^th^, 2020 – Jun 15^th^, 2020	Closing of all bars and restaurants
	Oct 13^th^, 2020 – May 11^th^, 2021	Closing all bars and restaurants, reopening of outside seating	Nov 1^st^, 2020 – Mar 25^th^, 2021	Closing of all bars and restaurants
Shops	Mar 17^th^, 2020 – Jun 1^st^, 2020	Preventive measures (distance, hand sanitising, visitor restrictions) to visit shops	Mar 17^th^, 2020 – May 11^th^, 2020	Closing of all non-essential shops
	Dec 15^th^, 2020 – Apr 28^th^, 2021	Closing of all non-essential shops, essential shops close at 20:00h		
Group size restrictions	Mar 23^rd^, 2020 – Jun 1^st^, 2020	Maximum of 2 people, except same household		
Wearing masks	Jun 1^st^ 2020 – Dec 31^st^ 2021	Mandatory in public transportation	Mar 17^th^, 2020 – May 11^th^, 2020	Mandatory in public spaces, from the age of 12 years and older
	Dec 1^st^ 2020 – Jun 26^th^ 2021	Mandatory in public buildings		
	Nov 8^th^ 2021 – Dec 31^st^ 2021	Mandatory in public buildings		

## Incidence of INS in the Netherlands

**Fig. 1:**
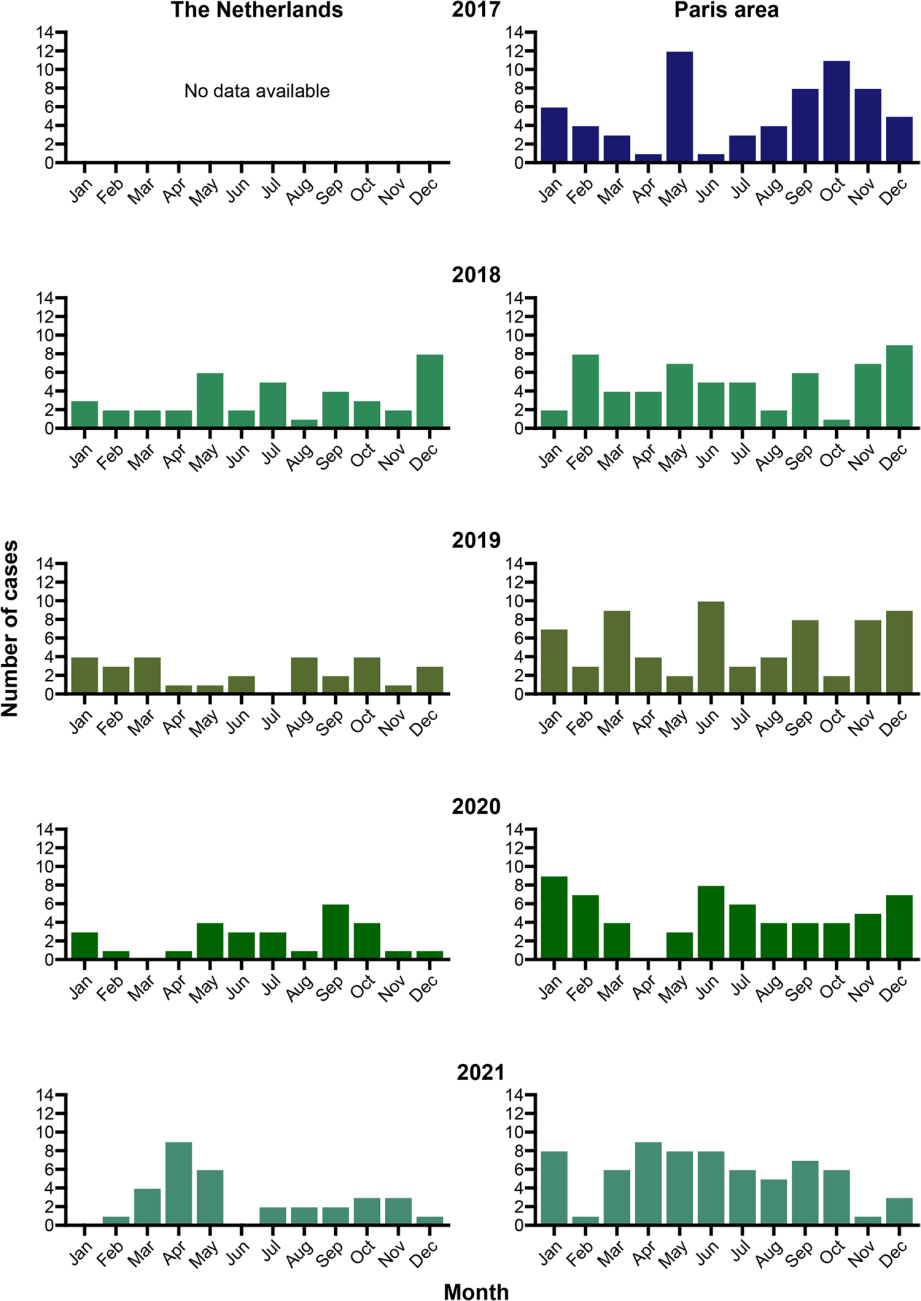
Monthly numbers of newly diagnosed INS in children in the a. Netherlands and b. the Paris area between January, 2017 and December, 2021. During the peak of the coronavirus pandemic in March, 2020, no new cases were presented

**Fig. 2:**
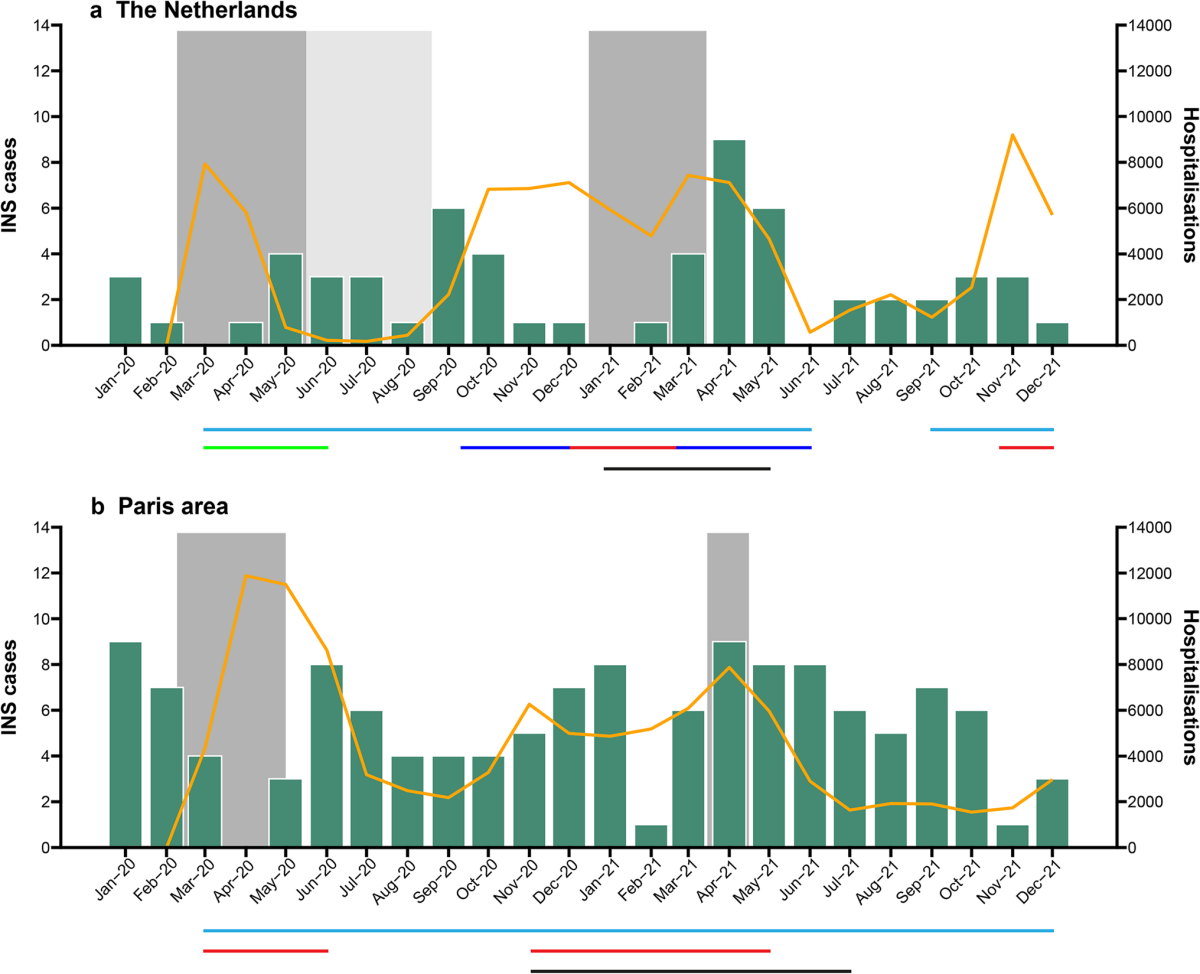
Close-up of the Covid-19 period 2020–2021 with the number of new cases (left axis) and indication of lockdown measures (grey blocks and coloured line below X-axis) and Covid-19-related hospitalisations (30-day average; right axis) in **a**. the Netherlands and **b**. Paris area. Grey blocks = Full school closures; Light grey blocks = Partial school closures. Light blue line = fundamental hygiene rules; Green line = “Intelligent” lockdown (Bars and restaurants closed, schools and sports events closed, non-essential shops opened, group size restrictions); Blue line = Partial lockdown (Bars and restaurants closed, non-essential shops opened, group size restrictions); Red line = Complete lockdown (Non-essential shops closed); Black line = Curfew

Figure 1a shows the monthly new cases of INS between 2018 and 2021. Overall incidence of INS in children in the Netherlands was 1.21 (95% CI 1.00–1.43) per 100,000 children per year (Table 3a). During the Covid-19 pandemic, incidence was lower (1.14, 95% CI 0.88–1.43) than before the pandemic (1.25, 95% CI 0.97–1.56), but this was not significant (*p =* 0.61). Overall incidence was higher in boys (*p =* 0.011) and in children under the age of 7 or under 12 (*p* < 0.001). When the schools were closed, incidence was significantly lower (0.53, 95% CI 0.15–0.96 vs. 1.31, 95% CI 1.07–1.56, *p =* 0.017). Other lockdown measures had no effect on incidence (Table 3a). When looking at absolute numbers, no cases occurred during the peak of the first (March, 2020) and second (January, 2021) wave, whereas there was an increase in cases when schools reopened after the summer holidays (September, 2020) and after the second lockdown (March, 2021) (Fig. 2a).

**Table 3:** Incidence of INS in a. the Netherlands and b. the Paris area, France, before and during the Covid-19 pandemic and during each lockdown period

a. The Netherlands	Reported cases, n	True cases^a^, n	95% CI	Population at risk^b^, n	Time, months	Incidence, n per 100,000	95% CI	*p* value^c^
Incidence 2018–2021	128	142	117–168	2,936,425	48	1.21	1.00–1.43	
Before Covid-19	73	80	61–100	2,936,425	26	1.25	0.97–1.56	0.61
During Covid-19	55	61	38–69		22	1.14	0.88–1.43	
Overall								
* Sex*								
Boys	80	88	69–110	1,504,400	48	1.47	1.15–1.82	0.011
Girls	48	53	39–70	1,432,025	48	0.93	0.68–1.22	
* Age*								
< 7 years	84	93	74–115	1,044,899	48	2.24	1.77–2.75	< 0.001
≥ 7 years	44	49	35–63	1,891,526	48	0.64	0.46–0.84	
< 12 years	112	124	102–150	1,971,304	48	1.58	1.29–1.91	< 0.001
≥ 12 years	16	18	10–26	965,121	48	0.45	0.25–0.69	
During Covid-19 pandemic								
* Lockdown*								
No lockdown	100	110	88–134	2,936,425	36	1.25	1.00–1.52	0.47
Any lockdown	28	31	20–43		12	1.06	0.67–1.48	
* Lockdown, specified*								
First lockdown	5	6	1–11	2,936,425	3	0.76	0.15–1.52	
Second lockdown	20	22	13–33		7	1.28	0.74–1.93	
Third lockdown	4	4	1–9		2	0.89	0.22–1.87	
*School/Sport*								
Open	121	135	110–161	2,936,425	42	1.31	1.07–1.56	0.017
Closed	7	8	2–14		6	0.53	0.15–0.96	
* Wearing masks*^*d*^								
Not mandatory	13	16	8–27	965,121	37	0.45	0.22–0.74	> 0.99
Mandatory in public buildings	4	5	1–11		11	0.48	0.11–1.00	
b. Paris area, France	Reported cases, n	True number of cases^a^, n	95% CI	Population at risk^b^, n	Time, months	Incidence, n per 100,000	95% CI	*p* value^c^
Incidence 2017–2021	324	324	290–359	2,516,400	60	2.58	2.30–2.85	
Before Covid-19 pandemic	211	211	184–239	2,516,400	38	2.64	2.30–3.00	0.54
During Covid-19 pandemic	113	113	94–134		22	2.44	2.04–2.90	
Overall								
* Sex*								
Boys	215	215	186–246	1,280,466	60	3.36	2.91–3.84	< 0.001
Girls	109	109	88–130	1,235,934	60	1.76	1.42–2.10	
* Age*								
< 7 year	246	246	215–277	957,252	60	5.13	4.49–5.79	< 0.001
≥ 7 year	78	78	62–95	1,559,148	60	1.00	0.80–1.22	
< 12 year	299	299	266–333	1,747,578	60	3.43	3.04–3.81	< 0.001
≥ 12 year	25	25	16–35	771,822	60	0.65	0.41–0.91	
* During Covid-19 pandemic*								
* Lockdown*								
No lockdown	311	311	277–348	2,516,400	56.5	2.63	2.34–2.94	0.19
Any lockdown	13	13	6–21		3.5	1.76	0.82–2.86	
* Lockdown, specified*								
1st lockdown	4	4	1–8	2,516,400	2	0.97	0.24–1.91	0.11
2nd lockdown	9	9	4–15		1.5	2.85	1.27–4.77	
* Schools*								
Open	320	320	286–356	2,516,400	58	2.63	2.35–2.93	0.049
Closed	4	4	3–8		2	0.94	0.24–1.91	
* Wearing masks*								
≥ *12 years*								
Not mandatory	18	18	11–26	771,822	42	0.67	0.41–0.96	0.99
Mandatory in public space and schools	7	7	2–12		18	0.60	0.17–1.04	
≥ *6 years*								
Not mandatory	87	87	70–105	1,719,198	46	1.33	1.05–1.59	0.72
Mandatory in public space and schools	24	24	15–34		14	0.75	1.19–1.79	

^a^True cases and corresponding 95% CIs were calculated using a Monte Carlo simulation (*n* = 1000) based on the response rate (90%) and reported cases. See Methods for more details

^b^Population at risk is defined as all children aged 1–16 years living in the Netherlands at January 1^st^ of 2021 (source: *Centraal Bureau voor de Statistiek*)

^c^By proportional z-test for incidence

^d^Wearing masks only affected children aged 12 years or older and therefore the denominator is the population ≥ 12-year-olds

^e^Population at risk is defined as all children aged 1–16 years living in the Paris area at January 1^st^ of 2021 (source: *Institute Nationale de la Statistique et Études Économiques*)

## Incidence of INS in the Paris area

Figure 1b shows the monthly new cases of INS between 2017 and 2021. Overall incidence of INS in children in the Paris area was 2.58 (95% CI 2.30–2.85) per 100,000 children per year (Table 3b). Similar to the Netherlands, incidence in boys (*p* < 0.001) and young children (*p* < 0.001) was higher. During the Covid-19 pandemic, the incidence of INS in children was 2.64 (95% CI 2.04–2.90), which was comparable to before the pandemic (2.44, 95% CI 2.30–3.00, *p =* 0.54). When the schools were closed, incidence was significantly lower (0.94, 95% CI 0.24–1.91 vs. 2.63 95% CI 2.35–2.93, *p =* 0.049). Subsequent lockdown measures – when schools stayed open – had no effect on incidence (Table 3b). There was an increase in cases when schools reopened in June 2020, while during peaks of hospital admissions for COVID-19 (April 2020), no case of INS was reported in the Paris area (Fig. 2b).

## Discussion

The results of this observational study in the Netherlands and the Paris area in France show that incidence of INS was lower when schools were closed and lockdown measures were at their maximum. There was no difference between the period before and during the Covid-19 pandemic in both countries.

It has been assumed that incidence of INS has been stable over the decades [4]. A recent epidemiological study in a multi-ethnic population of Atlanta, United States of America, showed comparable incidence rates over three different 5-year periods [21]. However, during the Covid-19 pandemic, the investigators of the LEARNS and NEPHROVIR-3 study noticed a drop in new onset INS. Based on the theory that viral infections precede or trigger INS onset [14, 15], we hypothesised that the Covid-19 pandemic could impact INS incidence. Since the start of the Covid-19 pandemic, case reports of new onset of INS during SARS-CoV-2 infection have been published both in adults and children [22–24], but also reports that viral infections were significantly reduced [25–30]. Therefore, we conducted two independent, retrospective population-based studies in two different European regions. Surprisingly, we did not find a difference in incidence between the before and during pandemic INS cohorts in either region. This is in line with findings from Southern California where the number of new cases the year before the pandemic was similar to that of the first year of the pandemic [17]. No higher incidence during the repeated peaks or waves of Covid-19 related hospitalisations – and thus, SARS-CoV-2 burden – was observed. On the contrary, new cases were absent. This may suggest that SARS-CoV-2 is not a specific trigger for INS onset, which was also observed in a recent Italian retrospective study [31] and systematic review of literature [32]. However, it must be noted that the Covid-19 burden in children was low, especially before the emergence of the Omicron variant.

Despite the low burden of SARS-CoV-2 infection in children [33], they were severely affected by the implementation of lockdown measures. The effect of lockdown was largest in the Paris area and when schools were closed. Differences in the severity of the lockdown measures between the countries may explain the difference in the magnitude of the effect of lockdown on the INS incidence. Right when the pandemic hit, social distancing and fundamental hygiene measures were implemented by the governments of both countries. However, considerable differences in the measures existed between countries, but also between subsequent lockdowns. During the first lockdown in France, the measures were stricter than those that followed in the second and third waves of the pandemic. Schools were closed from March 17^th^ until May 11^th^, 2020, which led to an immediate drop in incidence in March/April 2020. When schools reopened, children of 6 years and older had to wear face masks. Also, strict travel restrictions and time outside the house as well as closing of all non-essential businesses were implemented during first lockdown. This is in contrast to the Netherlands, where schools were closed from March to June, 2020, but businesses were still open, albeit with hygiene measures. Moreover, although discouraged, people were free to travel throughout the country and spend their time outside. On the other hand, schools in the Netherlands were repeatedly closed for longer periods of time. When comparing the three successive lockdowns, there was an increase in the number of new cases, which may reflect the gradual release in the strictness of each lockdown.

Our findings, combined with data on viral infections from the literature, support our hypothesis that the onset of INS is potentially driven by a (viral) infection. This is based on several observations. First, incidence in both regions was lower when schools were closed. The closing of schools seriously impacted the number of peer interactions and/or encounters between children, while working from home, travel restriction and curfew reduced contacts among adults. This did not only contain the spread of SARS-CoV-2, but also resulted in a rapid decrease in incidence of other virus-transmitted infections within weeks [34–36]. This is thought to be the result of social distancing (physical distance, fewer encounters, school closings), increasing barriers (face masks), and increased awareness for hygiene (washing hands, the use of disinfectants). Our results indicate that wearing face masks had little to no impact on INS incidence, which is probably explained by the fact that in both regions only children of 12 years or older were obliged to wear a mask in public spaces, including schools (in Paris, the measure was later extended to include all children of 6 years and older). Since INS is a disease that primarily manifests itself in young children with a significantly higher incidence in children < 7 years of age, the children who were impacted are only a small proportion of INS patients and thus had little influence on overall incidence. But even within the age groups that had to wear facemasks, no difference was found.

Second, the impact of reduced viral transmission by minimising social encounters on INS incidence is further underscored by our observation that when the latest school restrictions were lifted in in the Netherlands, this led to a marked increase in new onset INS in the three months following. Almost simultaneously, a peak in respiratory syncytial virus (RSV) occurred in the Netherlands and France [37]. Literature shows that with the phased reduction of the lockdown, different viruses showed different tendencies of re-emerging. There was a steep increase in respiratory viruses after the reopening of schools and public places [38] and after the obligation to wear a facemask was released [39]. In our study, however, there was no effect of wearing facemasks.

Multiple studies have shown that the rate of infection-related hospitalisations in children significantly decreased in the weeks following the first lockdown measures [25–28, 30]. Although an altered pattern in healthcare-seeking behaviour may have played a role, hospitalisation rates for epilepsy, urinary tract infections or other, more serious health conditions did not change. As INS does not resolve spontaneously, it is unlikely that avoidance of medical care out of fear of contracting Covid-19 is of influence here.

Two studies showed trends towards lower relapse rates of INS during the Covid-19 pandemic [18, 19], while one did find a significant lower relapse rate [17]. We did not include relapse rate in our study, since a substantial proportion of children was included in one of the RCTs (either LEARNS or NEPHROVIR-3) which could have biased the results. Although the association between relapses and triggering infections has been more clearly established for the occurrence of relapses than first onset INS [40–42], earlier studies have shown that children with first onset INS had a higher geno- and seroprevalence of EBV than controls [14]. Another study highlighted an association between enterovirus infection and childhood INS onset [15]. This again emphasises the potential role that viruses may play in triggering nephrotic syndrome. Whether this effect is direct or indirect (“second hit”) through a malfunctioning immune response, remains to be elucidated [13].

In addition, a previous dynamic epidemiological study has demonstrated time and space clustering of childhood INS cases in the Paris area [6], arguing for the role of one (or more) environmental triggering factor(s), yet to be identified. Besides the virus hypothesis, environmental pollution may also be a triggering factor. Air pollution has been associated with an increased risk of INS in children in Taiwan [43]. During the spring 2020 lockdown, measures to limit the spread of Covid-19 sharply reduced activities. A major decrease in air pollution levels was observed in metropolitan France and led to a reduction in NO_2_ and PM concentrations [44]. Again, whether air exposure may have a direct or indirect effect on disease triggering is currently unknown, but the role of other (unknown) environmental factors might have also been modified with lockdowns.

This study has some limitations. Although new cases were prospectively reported to the Dutch study team, the additional survey identified ten more first onset INS patients. The response rate was high, still it was not 100%. New onset INS cases could have been missed, which may have underestimated the incidence. We tried to overcome this by estimating the true number of cases using a Monte Carlo simulation, but it is unknown in which month cases were missed. On the other side, there was a 100% response rate in Paris and this study found a similar change in pattern. Yet, these two independent cohort studies were able to confirm the results of one another. Another limitation was that we could only use descriptive analyses due to the limited amount of data. The low number of cases prevented us from doing extensive analyses to identify an association between the effect of lockdown, lag time (time between implementation of a lockdown measure and its effect) or holidays on INS incidence. Furthermore, the retrospective design prevented us from obtaining samples for antigenic or serological assays to detect concomitant viral infections. Also, because of a high prevalence of asymptomatic forms of SARS-CoV-2 infection in children and a dramatic heterogeneity of testing policies over time, in the vast majority of patients there was no information on whether they had been recently infected with SARS-CoV-2 in the days or weeks preceding INS onset in the electronic patient file. Therefore, we cannot conclude that SARS-CoV-2 does not trigger INS with 100% certainty.

In conclusion, our results suggest a link between INS onset and viral infections in children, although COVID-19 does not appear to be a specific trigger. A significant decrease in incidence was observed in periods when child-to-child interaction was minimised by closing schools and thereby reducing viral transmission in two independent European cohorts. This enabled us to confirm our findings. Since incidence before and during the pandemic was not significantly different, we suggest that a more or less similar number of children are at risk for developing INS over large periods of time, but that in the absence of a (viral) trigger, the disease does not manifest itself. When the trigger remerges, the incidence peaks afterwards. This has been shown in a large epidemiological study in the Paris area (lower incidence in summer) [6] and in a small Japanese study (significantly higher number of new cases in autumn) [45]. Viruses as triggers for INS onset deserve our attention for future studies. With the help of multinational, prospective registry studies of new onset INS, valuable information about trends in incidence and associated factors could be added to the field of paediatric nephrology – especially since INS is a rare disease. Gaining insight into the pathogenesis of INS could provide researchers and clinicians new directions for future research, prevention of the disease, and targeted therapy.

## Supplementary information

Below is the link to the electronic supplementary material.

Graphical abstract (PPTX 202 KB)

Supplementary file 1 (PDF 628 KB)

## Data Availability

The datasets generated during and/or analysed during the current study are available from the corresponding author on reasonable request.
