# Supplementary material for: Incidence of idiopathic nephrotic syndrome during the Covid-19 pandemic in the Paris area (France) and in the Netherlands
Source: Pediatr Nephrol. 2023 May 16;38(11):3681–92. doi: 10.1007/s00467-023-06006-9 (PMC10186275; doi:10.1007/s00467-023-06006-9)
Supplement: Supplementary file 1 — Graphical abstract (PPTX 202 KB) [file 467_2023_6006_MOESM1_ESM.pptx]

## Slide 1
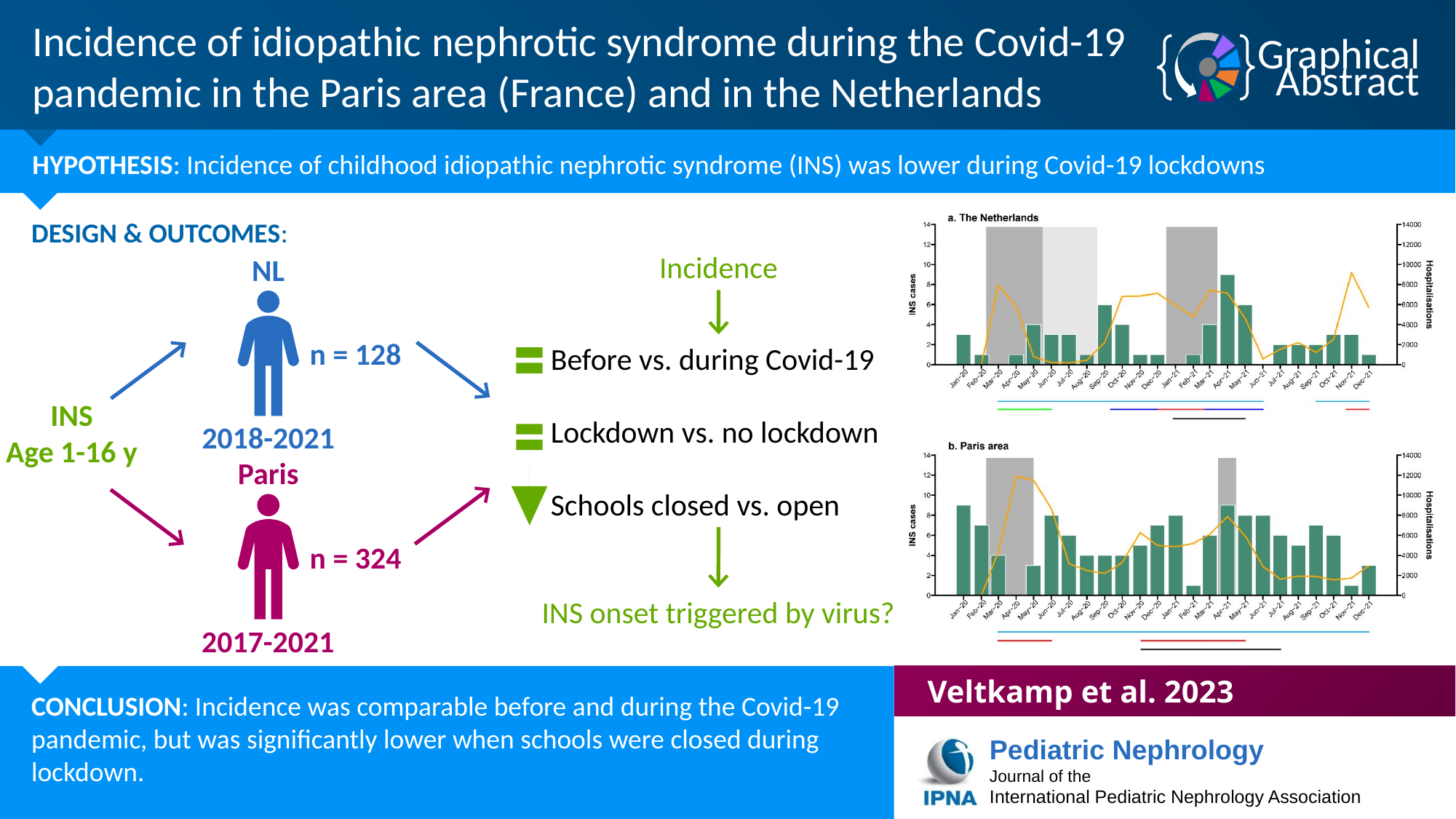

Incidence of idiopathic nephrotic syndrome during the Covid-19 pandemic in the Paris area (France) and in the Netherlands
HYPOTHESIS: Incidence of childhood idiopathic nephrotic syndrome (INS) was lower during Covid-19 lockdowns
DESIGN & OUTCOMES:
Incidence
NL
n = 128
Before vs. during Covid-19
Lockdown vs. no lockdown
Schools closed vs. open
INS
Age 1-16 y
2018-2021
Paris
n = 324
INS onset triggered by virus?
2017-2021
Veltkamp et al. 2023
CONCLUSION: Incidence was comparable before and during the Covid-19 pandemic, but was significantly lower when schools were closed during lockdown.
