# Supplementary material for: Incidence of idiopathic nephrotic syndrome during the Covid-19 pandemic in the Paris area (France) and in the Netherlands
Source: Pediatr Nephrol. 2023 May 16;38(11):3681–92. doi: 10.1007/s00467-023-06006-9 (PMC10186275; doi:10.1007/s00467-023-06006-9)
Supplement: Supplementary file 2 — Supplementary file 1 (PDF 628 KB) [file 467_2023_6006_MOESM2_ESM.pdf]

Supplementary Material

to:

Incidence of idiopathic nephrotic syndrome during the Covid-19 pandemic in the Paris area (France) and in the Netherlands

Floor Veltkamp, MD[1*‡], Victoire Thenot, MD[2*], Carlijn Mussies, MD[1], Bas van Lieshout, MD[1], Hessel Peters-Sengers, PhD[3,4], Jesper Kers, MD PhD[5,6,7], Djera H. Khan, MD[4], Julian Hogan, MD PhD[2], Sandrine Florquin, MD PhD[4], Antonia H.M. Bouts, MD PhD[1*], Claire Dossier, MD PhD[2*], on behalf of the NEPHROVIR network and the LEARNS consortium.

**These authors contributed equally to the study.*

1. Amsterdam University Medical Centers, University of Amsterdam, Emma Children's Hospital, Department of Pediatric Nephrology, Amsterdam, the Netherlands.
2. Department of Pediatric Nephrology, Robert-Debré Hospital, APHP, Paris, France.
3. Amsterdam University Medical Centers, University of Amsterdam, Center for Experimental and Molecular Medicine, Amsterdam, the Netherlands.
4. Department of Epidemiology and Data Science, VU University Amsterdam, Amsterdam University Medical Center, Amsterdam, Netherlands
5. Amsterdam University Medical Centers, University of Amsterdam, Department of Pathology, Amsterdam, the Netherlands.
6. Department of Pathology, Leiden University Medical Center, Leiden, the Netherlands
7. Van 't Hoff Institute for Molecular Sciences, University of Amsterdam, Amsterdam, the Netherlands

‡Corresponding author: Floor Veltkamp, MD, Amsterdam University Medical Centers, University of Amsterdam, Emma Children's Hospital, Department of Pediatric Nephrology, Meibergdreef 9, 1109 AZ, Amsterdam, the Netherlands

Appendix 1 List of hospitals in the Netherlands with a paediatric ward that were contacted to complete the survey. Some hospitals had multiple locations and were counted individually. Fourteen hospitals were participating in the LEARNS study.

Hospital	City	Completed	LEARNS
Wilhelmina Ziekenhuis	Assen	X	
Scheper Ziekenhuis	Emmen	X	
Bethesda Ziekenhuis	Hoogeveen	X	
Koningin Beatrixziekenhuis	Winterswijk	X	
Ommelander Ziekenhuis Groningen	Scheemda	X	
Treant, ziekenhuislocatie Refaja	Stadskanaal	X	
Martini Ziekenhuis	Groningen	X	
UMCG / Beatrix Kinderkliniek	Groningen	X	X
Ziekenhuis Nij Smellinghe	Drachten	X	
Ziekenhuis Tjongerschans	Heerenveen	X	
Antonius Ziekenhuis	Sneek		
Medisch Centrum Leeuwarden	Leeuwarden	X	X
Ziekenhuisgroep Twente	Hengelo		
Deventer Ziekenhuis	Deventer	X	X
Medisch Spectrum Twente	Enschede	X	X
Medisch Spectrum Twente	Oldenzaal		
Medisch Spectrum Twente	Haaksbergen	X	
Röpcke-Zweers Ziekenhuis, *now Saxenburgh MC*	Hardenberg		
Isala Kliniek	Meppel	X	
Isala Kliniek	Zwolle	X	X
Ziekenhuis Ommen	Ommen		
Gelre Ziekenhuis	Apeldoorn	X	
Gelre Ziekenhuis	Zutphen	X	
Ziekenhuis Rijnstate	Arnhem	X	
Slingeland Ziekenhuis	Doetinchem	X	
Ziekenhuis Gelderse Vallei	Ede	X	
Ziekenhuis Gelderse Vallei	Barneveld	X	
Ziekenhuis St. Jansdal	Harderwijk	X	
Canisius Wilhelmina Ziekenhuis	Nijmegen	X	
Radboudumc / Amalia Kinderziekenhuis	Nijmegen	X	X
Ziekenhuis Rivierenland	Tiel	X	
Meander MC	Amersfoort	X	
Meander MC	Baarn	X	
Diakonessenhuis	Utrecht		
St. Antonius Ziekenhuis	Nieuwegein	X	
St. Antonius Ziekenhuis	Utrecht	X	
UMCU / Wilhemina Kinderziekenhuis	Utrecht		
Jeroen Bosch Ziekenhuis	‘s Hertogenbosch		
Catharina Ziekenhuis	Eindhoven	X	
Máxima Medisch Centrum	Veldhoven	X	
Amphia Ziekenhuis	Breda	X	X

Hospital	City	Completed	LEARNS
St. Anna Ziekenhuis	Geldrop	X	
Elkerliek Ziekenhuis	Helmond	X	
Elisabeth-TweeSteden Ziekenhuis	Tilburg	X	
Maasziekenhuis Pantein	Beugen	X	
Bravis Ziekenhuis	Bergen op Zoom	X	
Ziekenhuis Bernhoven	Oss/Uden	X	
Zuyderland Ziekenhuis	Brunssum	X	
Zuyderland Ziekenhuis	Heerlen	X	
Zuyderland Ziekenhuis	Kerkrade	X	
Zuyderland Ziekenhuis	Sittard-Geleen		
Laurentius Ziekenhuis	Roermond	X	
Sint Jans Gasthuis	Weert	X	
MUMC +	Maastricht		X
VieCuri Medisch Centrum	Venlo		
Admiraal de Ruyter Ziekenhuis	Vlissingen		
Admiraal de Ruyter Ziekenhuis	Goes		
Zorgsaam	Terneuzen		
Flevoziekenhuis / Kinderkliniek	Almere	X	
Noordwest Ziekenhuisgroep	Alkmaar	X	X
Noordwest Ziekenhuisgroep	Den Helder	X	
Amstelland Ziekenhuis	Amstelveen	X	
Amsterdam UMC / Emma Kinderziekenhuis	Amsterdam	X	X
BovenIJ Ziekenhuis	Amsterdam	X	
OLVG locatie Oost	Amsterdam		
OLVG locatie West	Amsterdam	X	
Amsterdam UMC, locatie VUmc	Amsterdam	X	
TerGooi Ziekenhuis	Blaricum	X	
Rode Kruisziekenhuis	Beverwijk	X	
Spaarne Gasthuis	Haarlem	X	X
Dijklander Ziekenhuis	Hoorn	X	
Dijklander Ziekenhuis	Purmerend	X	
Zaans Medisch Centrum	Zaandam	X	
Alrijne Ziekenhuis	Leiderdorp	X	
Franciscus Gasthuis	Rotterdam	X	
Franciscus Vlietland	Schiedam		
Van Weel-Bethesda Ziekenhuis	Dirksland	X	
Reinier de Graaf Gasthuis	Delft	X	
MC Haaglanden, Bronovo	The Hague	X	
Haga Ziekenhuis / Juliana Kinderziekenhuis	The Hague	X	X
Albert Schweitzer Ziekenhuis	Dordrecht		
Beatrixziekenhuis - Rivas Zorggroep	Gorinchem		
Groene Hart Ziekenhuis	Gouda	X	
LUMC / Willem- Alexander Kinderziekenhuis	Leiden	X	X
Erasmus MC / Sofia Kinderziekenhuis	Rotterdam	X	X
Ikazia Ziekenhuis	Rotterdam	X	
Maasstad Ziekenhuis	Rotterdam		
IJsselland Ziekenhuis	Rotterdam	X	

Hospital	City	Completed	LEARNS
**Langeland Ziekenhuis**	Zoetermeer	X	

Online Resource 2 – Survey sent to all hospitals with a paediatric ward in the Netherlands.

PART I – Resondent details
**1. Which hospital/medical centre are you currently affiliated to?** *If there are multiple hospitals/centres: please fill in all hospital/centres you have been working with corresponding years.*
**Definition of INS:**<br><br>First episode idiopathic nephrotic syndrome is defined by the following clinical features:<br>- Oedema<br>- Proteinuria (>200 mg/mmol creatinine or >40 mg/m^2 per hour)<br>- Hypoalbuminaemia (<25 g/L)<br>- No secondary or congenital cause leading to nephrotic syndrome
**3. Did you have any case of first episode of INS in either 2018, 2019, 2020, or 2021?**<br>□ Yes<br>□ No
**PART II – Case details**
**4. Date of first presentation of INS**. *Enter month and year.*<br>01-MMM-YYYY
**5. To which hospital presented the patient with first episode INS.**<br>_____________________________
**6. Age of the patient with first episode INS.**<br>_____ years
**7. Sex of the patient with first episode INS.**<br>□ Female<br>□ Male
**8. If presented in either 2020 or 2021, did the patient with first episode INS had a recent SARS-CoV-2 infection?**<br>□ No<br>□ Yes
**9. Do you have any additional remarks regarding the case?**<br>____
